# Uremic Toxins Affecting Cardiovascular Calcification: A Systematic Review

**DOI:** 10.3390/cells9112428

**Published:** 2020-11-06

**Authors:** Jana Holmar, Sofia de la Puente-Secades, Jürgen Floege, Heidi Noels, Joachim Jankowski, Setareh Orth-Alampour

**Affiliations:** 1 Institute for Molecular Cardiovascular Research (IMCAR), RWTH Aachen University, University Hospital Aachen, 52074 Aachen, Germany; jholmar@ukaachen.de (J.H.); sdelapuentes@ukaachen.de (S.d.l.P.-S.); hnoels@ukaachen.de (H.N.); 2 Division of Nephrology, RWTH Aachen University, University Hospital Aachen, 52074 Aachen, Germany; jfloege@ukaachen.de; 3 Department of Biochemistry, Cardiovascular Research Institute Maastricht (CARIM), Maastricht University, 6229 ER Maastricht, The Netherlands; 4 Department of Pathology, Cardiovascular Research Institute Maastricht (CARIM), Maastricht University Medical Centre, Maastricht University, 6229 ER Maastricht, The Netherlands

**Keywords:** cardiovascular calcification, uremic toxins, chronic kidney disease, cardiovascular disease

## Abstract

Cardiovascular calcification is highly prevalent and associated with increased morbidity in chronic kidney disease (CKD). This review examines the impact of uremic toxins, which accumulate in CKD due to a failing kidney function, on cardiovascular calcification. A systematic literature search identified 41 uremic toxins that have been studied in relation to cardiovascular calcification. For 29 substances, a potentially causal role in cardiovascular calcification was addressed in in vitro or animal studies. A calcification-inducing effect was revealed for 16 substances, whereas for three uremic toxins, namely the guanidino compounds asymmetric and symmetric dimethylarginine, as well as guanidinosuccinic acid, a calcification inhibitory effect was identified in vitro. At a mechanistic level, effects of uremic toxins on calcification could be linked to the induction of inflammation or oxidative stress, smooth muscle cell osteogenic transdifferentiation and/or apoptosis, or alkaline phosphatase activity. For all middle molecular weight and protein-bound uremic toxins that were found to affect cardiovascular calcification, an increasing effect on calcification was revealed, supporting the need to focus on an increased removal efficiency of these uremic toxin classes in dialysis. In conclusion, of all uremic toxins studied with respect to calcification regulatory effects to date, more uremic toxins promote rather than reduce cardiovascular calcification processes. Additionally, it highlights that only a relatively small part of uremic toxins has been screened for effects on calcification, supporting further investigation of uremic toxins, as well as of associated post-translational modifications, on cardiovascular calcification processes.

## 1. Introduction

Approximately 13% of the population suffers from chronic kidney disease (CKD) globally, and about half of the patients with CKD stages 4-5 also suffer and eventually die from cardiovascular disease (CVD) [1,2]. The presence of cardiovascular calcifications highly increases cardiovascular risk [3,4]. Cardiovascular calcification increases with age in the general population [5], but is massively accelerated [6] and highly frequent in patients with CKD. Reports indicate up to 70% of CKD patients presenting abdominal aortic calcification as detected by plain X-rays [7,8] and even 88% of a cohort of 20–30-year old patients in CKD stage 5D already displayed vascular calcifications [9]. Furthermore, up to 100% of patients in CKD stage 5D displayed calcification in the aortic valve, mitral annulus or mitral valve as imaged by echocardiography [8].

Consequences of cardiovascular calcification include decreased arterial elasticity, an increase in pulse wave velocity, a decrease in coronary artery perfusion, aortic valve stenosis, progression of left ventricular hypertrophy as well as myocardial ischemia and failure [10,11]. These cardiovascular complications are among the main causes of death in CKD patients [2] and cardiovascular calcification heralds a poor prognosis in terms of cardiovascular morbidity in these patients [12,13]. In line, KDIGO (Kidney Disease: Improving Global Outcomes) CKD guidelines suggest to consider patients with advanced CKD (stages 3–5) and cardiovascular calcifications at the highest cardiovascular risk [14].

Cardiovascular calcification is an actively regulated and progressive process that, once initiated, tends to relentlessly increase in severity. The mechanisms underlying cardiovascular calcification involve the accumulation of calcium and phosphate in the extracellular matrix (ECM), the transdifferentiation of vascular smooth muscle cells (VSMC) to osteoblast/chondrocyte-like cells, increased VSMC apoptosis and reduced proliferation [15]. Furthermore, inflammation and oxidative stress have been reported as inducers of VSMC osteogenic transdifferentiation and calcification [16,17]. Additionally, cardiovascular calcification goes along with elastin degradation in the vascular wall, which is mediated by matrix metalloproteinases (MMPs). The enhanced elastin degradation increases the affinity of the ECM for calcium and promotes the growth of hydroxyapatite crystals along the elastic lamina and finally ECM mineralization [18]. Increased cardiovascular calcification in CKD is believed to result from a disturbed balance between inhibitors and inducers of calcification, but the exact mechanisms are not completely understood and are believed to be multifactorial [13].

Uremic toxins are defined as harmful substances that accumulate in the circulation of CKD patients due to the failing function of the kidney [19]. To date, over 140 uremic toxins have been described to be increased in CKD [20,21]. These can be divided into three groups according to their physicochemical characteristics [19]: (i) free, water-soluble, low molecular weight solutes (MW < 500 Da); (ii) water-soluble, middle-sized molecules (MW > 500 Da); (iii) protein-bound solutes. Serum levels of for example p-cresyl sulphate and indoxyl sulphate have been shown to be associated to cardiovascular outcome in CKD [22,23] and positively correlated with vascular calcification and stiffness in CKD [24,25,26]. This has raised the hypothesis that the accumulation of uremic toxins in CKD may contribute to the high prevalence of cardiovascular calcification in these patients. However, despite many comprehensive reviews on cardiovascular calcification in CKD [13,27], there are still many uncertainties about which uremic toxins and associated mechanisms affect cardiovascular calcification. As such information may reveal novel treatment modalities to reduce vascular calcification and could support a future improvement of removal techniques, this systematic review comprehensively analyzes available studies on the effect of uremic toxins on calcification processes.

## 2. Materials and Methods

### 2.1. Data Sources and Searches

**Figure 1:**
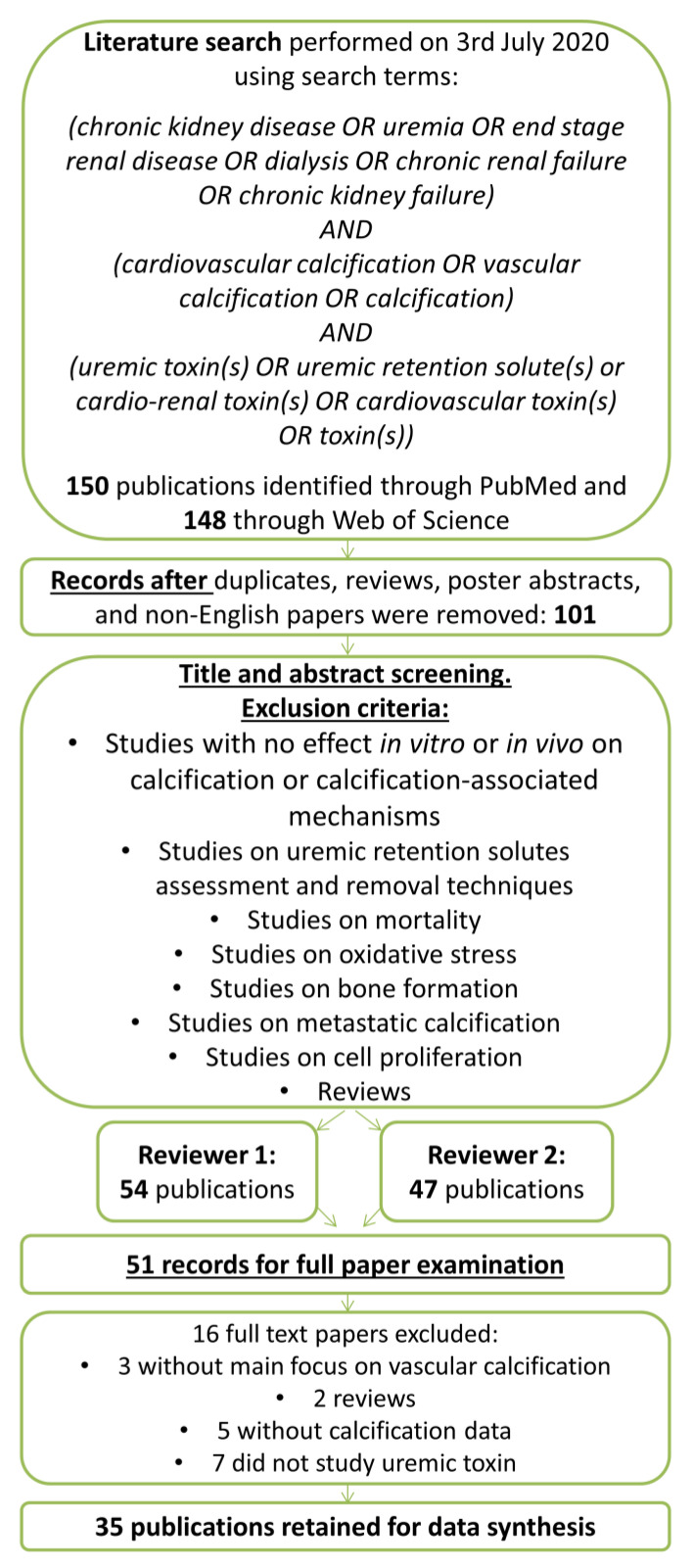
Flow diagram for original paper selection for the systematic review on uremic toxins and cardiovascular calcification.

The authors performed an advanced search in both PubMed and Web of Science to identify eligible studies on 3 July 2020. The search strategy is depicted in Figure 1. The following terms and conditions were used for the systematic literature search: chronic kidney disease OR uremia OR end stage renal disease OR dialysis OR chronic renal failure OR chronic kidney failure AND cardiovascular calcification OR vascular calcification OR calcification AND uremic toxin(s) OR uremic retention solute(s) OR cardio-renal toxin(s) OR cardiovascular toxin(s) OR toxin(s). Where indicated, additional literature on identified uremic toxins was added manually after checking the reference lists of the included studies or recent reviews, when required for a correct interpretation of findings. The Preferred Reporting Items for Systematic Reviews and Meta-Analyses (PRISMA) guidelines were followed while composing and writing the systematic review. The systematic review was registered in the PROSPERO database (CRD42016051604) on 16 November 2016 [28].

### 2.2. Study Selection Criteria

Two screeners independently identified and evaluated papers by the title and the abstract for inclusion or exclusion. The full articles and/or supplemental materials (tables and appendices) were reviewed before the inclusion/exclusion decision. Articles describing the influence of one or more uremic toxin(s) on cardiovascular calcification were included into the analyses. Duplicates, review papers, poster abstracts, and non-English papers were excluded. Papers were also excluded whose main focus was not cardiovascular calcification, which did not study uremic toxins, which described different uremic toxins assessment/removal techniques, or which studied mainly oxidative stress, bone formation, metastatic calcification, or cell proliferation. There were no restrictions on sample size or study duration defined by the authors. A third reviewer was consulted to achieve consensus in case of inconsistencies.

## 3. Results

### 3.1. Identification of Uremic Toxins Studied in the Context of Uremia and Cardiovascular Calcification

The search yielded 148 hits in Web of Science and 150 in PubMed. After removing duplicates, review papers, poster abstracts, and non-English papers, the titles, and abstracts of the remaining 101 papers were screened. Out of these, 51 papers were selected for full-text examination and 35 of those papers were identified as relevant for this systematic review. Within these, 41 substances with increased plasma concentration in CKD patients were studied in the context of uremia and cardiovascular calcification. A total of 12 substances were only investigated for a potential association with the extent of cardiovascular calcification in CKD patients (Table S1) and are not further discussed here. For the remaining 29 substances, a potentially causal role in cardiovascular calcification was addressed in in vitro or animal studies. These included 15 low molecular weight uremic toxins (Table 1); 12 middle molecular weight uremic toxins (Table 2) as well as indoxyl sulphate and p-cresyl sulphate as protein-bound uremic toxins (Table 3). Where indicated in Table 1, Table 2 and Table 3, additional studies on these substances were included after screening the reference lists of the included studies or recent reviews.

Applied methods for determining calcification extent included histochemical stainings (alizarin red and von Kossa), calcium content measurements using the o-cresolphthalein complexone method and analysis of alkaline phosphatase (ALP) activity. Additionally, to investigate potential underlying mechanisms, studies quantified effects on the expression of calcification inhibitors fetuin-A or osteopontin or analyzed a potential dysregulation of marker proteins and genes previously associated with calcification processes, as explained in more detail below.

### 3.2. Effect of the Identified Substances on Cardiovascular Calcification

**Figure 2:**
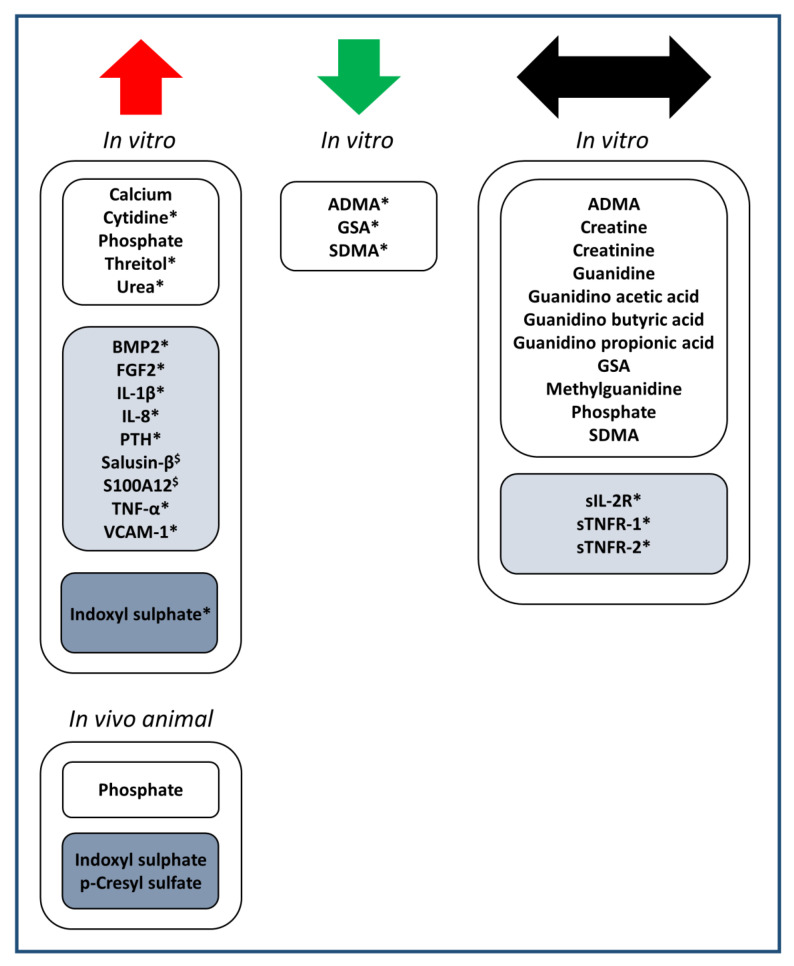
Overview of uremic toxins studied for potentially causal effects on vascular calcification in in vitro or animal studies. The effect on calcification is indicated with arrows in red (increased calcification), green (decreased calcification) or black (no effect). Multiple listing is possible in case of contrasting published findings. Uremic toxins are classified as low molecular weight (white boxes), middle molecular weight (light blue boxes) or protein-bound (dark blue boxes). * in pro-calcifying media; ^$^ in pro- as well as non-calcifying media.

The substances presented in Table 1, Table 2 and Table 3 were categorized according to their effect on cardiovascular calcification, being either inhibiting, inducing, or no effect, as visualized in Figure 2.

#### 3.2.1. Low Molecular Weight Substances, Increased in Blood in CKD (Table 1)

A high ambient phosphate concentration triggers calcification in a variety of situations, as shown in vitro in human VSMC [29,30,32,33,34], rat and mouse VSMC [35,36], rat aortic rings [33], as well as in aortas of rats subjected to 5/6 nephrectomy [35]. *Apo^−/−^* mice with 5/6 nephrectomy displayed aortic valve calcification 12 weeks after surgery both when fed a high phosphate diet as well as a control diet, though with increased calcium deposits detected upon high phosphate diet [36]. Additionally, elevated calcium levels triggered calcification in human VSMC in vitro as well as ex vivo studies with rat aortic rings [38,40].

Calcification of human VSMC could not be induced upon treatment with the guanidino compounds asymmetric dimethylarginine (ADMA), symmetric dimethylarginine (SDMA), guanidinosuccinic acid (GSA) or seven other guanidino compounds (creatine, creatinine, guanidine, guanidino acetic acid, guanidino butyric acid, guanidino propionic acid, methylguanidine), although ADMA, SDMA, and GSA could reduce human VSMC calcification in high inorganic phosphate media [29]. In contrast, adding the small molecules cytidine, urea, and threitol to human MSC incubated in pro-calcifying medium increased ALP activity and calcium content [41].

#### 3.2.2. Middle Molecular Weight Substances, Increased in Blood in CKD (Table 2)

Addition of bone morphogenetic protein 2 (BMP2) increased the calcium concentration in bovine VSMC cultured in calcifying medium [42]. Additionally, adding fibroblast growth factor 2 (FGF2), interleukin-1β (IL-1β), PTH or tumor necrosis factor alpha (TNF-α) to human MSC in pro-calcifying medium increased ALP activity and the calcium content, and upregulated osteoblast markers such as CBFA1/RUNX2, osteopontin, and osterix [41]. The pro-inflammatory cytokine TNF-α also increased calcium content [33,49] and ALP activity [48] in human VSMC in calcifying medium, as well as increased the calcium content of rat aortic rings in calcifying conditions [33]. Similarly, addition of vascular cell adhesion molecule-1 (VCAM-1) or the pro-inflammatory cytokine interleukin-8 (IL-8) to human VSMC in pro-calcifying medium increased calcification as shown by alizarin red staining and increased ALP activity (for VCAM-1) or calcium content (for IL-8) [30,48]. The RAGE ligand S100A12 induced calcification in mouse VSMC as revealed by alizarin red staining, which was linked to an increased generation of reactive oxygen species (ROS) as well as an increased mRNA expression of the phosphate co-transporter PIT-1 and the osteoblast markers BMP2 and CBFA1/RUNX2 [36]. Overexpression of the peptide salusin-β, shown to be increased in patients undergoing hemodialysis [47], increased calcification of human VSMC and rat aortic rings, not only in calcification media, but also in media without calcification stimulation, as demonstrated by calcium content, alizarin red, or von Kossa staining as well as ALP activity. In parallel, salusin-β increased expression of the osteogenic markers BMP2 and RUNX2, while decreasing expression of contractile SMC markers alpha-smooth muscle actin (α-SMA) and smooth muscle protein 22-alpha (SM22-α) in human VSMC [46]. In contrast, soluble cytokine receptors sTNFR-1 and -2 [48] and sIL-2R [48] did not increase ALP activity nor calcification extent in alizarin red staining when added to human VSMC in pro-calcifying medium.

#### 3.2.3. Protein-Bound Substances, Increased in Blood in CKD (Table 3)

In human VSMC, indoxyl sulphate increased calcium content when added to pro-calcifying incubation medium [30] and induced calcification as shown by alizarin red staining [30,51,52,53,54]. In parallel, it reduced the expression of α-SMA [30,51,54] and SM22-α [30], but upregulated CBFA1/RUNX2 [30,51,52,53,54], osteopontin [30,51,52,53], ALP, and BMP2 [30,51]. Rat aortic rings incubated in high phosphate media in the presence of indoxyl sulphate had increased calcium content and showed enhanced calcification in alizarin red staining [30]. Furthermore, administrating indoxyl sulphate to rats with hypertension [57,58], 5/6 nephrectomy [51] and adenine-induced CKD [59] increased aortic calcium content [59] and calcification as shown by von Kossa [57,58,59] or alizarin red staining [51]. An increased calcium content was also determined in aortas of adenine-induced CKD rats receiving p-cresyl sulphate and confirmed by von Kossa staining [59].

### 3.3. Mechanisms and Signalling Pathways Associating Uremic Toxins with Calcification

The calcium-phosphate metabolism is one of the key factors for initiation and progression of vascular calcification in CKD [60] and in vitro as well as ex vivo studies showed increased calcification upon increased levels of calcium [38,40] or inorganic phosphate [29,32,33,35]. In late stage CKD (CKD 4–5), most of CKD patients present with hyperphosphatemia [61,62]. On the other hand, in CKD patients, calcium metabolism is complex and the total body calcium levels vary over the different stages of CKD. In this regard, hypocalcemia is quite uncommon in CKD stage 3 and early stage 4, but more often observed in stage 5 as long as patients are not yet on dialysis [63,64].

**Figure 3:**
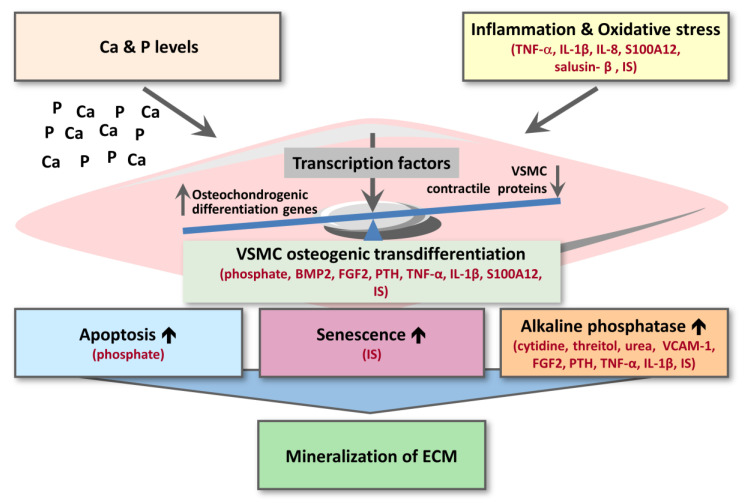
Scheme of uremic toxins affecting different regulatory steps of cardiovascular calcification. For details, we refer to the text. In red are shown the inducers of cardiovascular calcification. BMP2—bone morphogenetic protein 2; Ca—calcium; ECM—extracellular matrix; FGF2—fibroblast growth factor 2; IL-1β—interleukin-1 beta; IL-8—interleukin-8; IS—indoxyl sulphate; P—phosphate; PTH—parathyroid hormone; TNF-α—tumor necrosis factor-alpha; VCAM-1—vascular cell adhesion molecule-1; VSMC—vascular smooth muscle cell.

For the other substances from Table 1, Table 2 and Table 3, potential mechanisms underlying their effect on vascular calcification can be subdivided in: (i) inflammation and oxidative stress; (ii) VSMC osteogenic transdifferentiation; (iii) VSMC apoptosis or senescence; (iv) alkaline phosphatase activity (Figure 3). Osteogenic transdifferentiation is driven by the upregulation of transcription factors (e.g., CBFA1/RUNX2, MSX2, SOX9) controlling the expression of osteochondrogenic marker proteins (BMP2, osterix, osteocalcin, ALP, osteopontin) as well as the downregulation of smooth muscle contractile proteins (e.g., SM22-α, α-SMA, SMTN) (Figure 3) [65].

#### 3.3.1. Inflammation and Oxidative Stress

On the one hand, cardiovascular calcification seems to accelerate inflammation and oxidative stress [66]. On the other hand, systemic inflammation and oxidative stress have been identified as drivers of cardiovascular calcification in CKD patients. Thus, cardiovascular calcification and inflammation potentially form a vicious circle in CKD. For the substances in Table 1, Table 2 and Table 3, following links with inflammation were identified:

Phosphate (Table 1): Treatment of mouse VSMC with high phosphate medium induced ROS production and ROS-mediated expression of S100 proteins as pro-inflammatory RAGE ligands along with osteogenic marker expression and calcification. The phosphate co-transporter PIT-1, shown to be crucial for phosphate-induced calcification, was upregulated by high phosphate medium through involvement of the RAGE receptor, which could be blocked by anti-oxidant treatment [36].

ADMA, SDMA, and GSA (Table 1): Guanidino compounds constitute a large group of low molecular weight uremic toxins that are generated as a result of protein and amino acid metabolism. They can exert pro-inflammatory as well as anti-inflammatory effects, which may modulate the prevalence of CVD in patients with CKD [67]. Among investigated guanidino compounds, ADMA, SDMA, and GSA inhibited cardiovascular calcification in human VSMC in high inorganic phosphate media [29]. The underlying mechanisms still remain to be clarified, as ADMA, SDMA, and GSA exerted mainly pro-inflammatory effects, at least on leukocytes [29].

TNF-α, interleukin-1β, interleukin-8 and S100A12 (Table 2): Incubation of rat aortic rings as well as human VSMC with phosphate and additionally the pro-inflammatory cytokine TNF-α induced a higher increase in calcium content compared to incubation with high phosphate alone, linking inflammation and vascular calcification [33]. As underlying mechanism, TNF-α-induced VSMC changes towards an osteogenic phenotype were observed [33]. In line with this, incubation of pluripotent undifferentiated MSC with TNF-α as well as with the pro-inflammatory cytokine interleukin-1β (IL-1β) enhanced osteoblastic transdifferentiation and calcification [41], linking pro-inflammatory mediators to vascular calcification over osteogenic transdifferentiation. Additionally, the pro-inflammatory RAGE ligand S100A12 induced expression of osteoblast markers BMP2 and CBFA1/RUNX2 in mouse VSMC along with increased calcification, with ROS-induced PIT-1 expression revealed to be important in S100A12-induced calcification [36]. For the pro-inflammatory cytokine interleukin-8 (IL-8), increased calcification extent in human VSMC in pro-calcifying conditions was mechanistically linked to a downregulation of the calcification inhibitor osteopontin [30].

Salusin-β (Table 2): Overexpressing salusin-β, a small bioactive peptide that is increased in patients undergoing hemodialysis [47], enhanced calcification of human VSMC along with an increased expression of the NAD(P)H oxidase subunits p22 and p47 and enzyme NOX2, which are involved in ROS production. Inhibiting NAD(P)H oxidase or scavenging ROS reduced ALP activity and calcium deposition induced by salusin-β overexpression in VSMC. Additionally, in rat aortas, overexpression of salusin-β increased the expression of the NAD(P)H oxidase subunits and NOX2 as well as intensified the production of malondialdehyde and hydrogen peroxide as oxidative stress markers. Combined, the results suggested that the increase in vascular calcification by salusin-β is due to the activation of the NAD(P)H oxidase-ROS pathway [46].

Indoxyl sulphate and p-cresyl sulphate (Table 3). Treatment of hypertensive rats with the uremic toxin indoxyl sulphate not only induced vascular calcification [57,58], but also increased expression of oxidative stress markers 8-hydroxyl-2′-deoxyguanosine (8-OHdG) and malondialdehyde in aorta [55]. Calcified aortic regions of indoxyl sulphate-treated rats also displayed enhanced expression of p53 (a trigger of apoptosis as well as senescence), the cell cycle inhibitor p21, as well as prelamin A as hallmarks of VSMC senescence [55,58]. In human VSMCs in vitro, these effects were counteracted by anti-oxidant treatment [55]. Based on these findings, a role for indoxyl sulphate-induced oxidative stress in vascular calcification through effects on cell cycle arrest and VSMC senescence was suggested [55,57,58]. Furthermore, in vivo studies of rats with adenine-induced CKD and treated with indoxyl sulphate or p-cresyl sulphate showed, in parallel with increased vascular calcification, activation of inflammation (acute-phase response) and coagulation signaling pathways in the calcified aorta [59].

#### 3.3.2. VSMC Osteogenic Transdifferentiation

A link of increased calcification along with VSMC osteogenic transdifferentiation has been shown in vitro for phosphate (Table 1), BMP2, FGF2, PTH, TNF-α, IL-1β, and S100A12 (Table 2) as well as indoxyl sulphate (Table 3):

Phosphate (Table 1): Along with vascular calcification, a high concentration of phosphate induced a significant increase in CBFA1/RUNX2, BMP2 and osteopontin expression in mouse VSMC [36,37], with an important role for the RAGE receptor and oxidative stress [36]. This latter finding demonstrates a link between inflammation and VSMC osteogenic transdifferentiation induced by high phosphate, with (RAGE receptor-mediated) oxidative stress contributing to increased expression of the phosphate co-transporter PIT-1 as well as RUNX2 upon high-phosphate treatment of VSMC, and with pharmacological inhibition of the phosphate co-transporters PIT-1 and PIT-2 interfering with high phosphate-induced calcification of VSMC [36]. Similarly, high phosphate levels significantly increased MSX2, SOX9, CBFA1/RUNX2, BMP2, and osteopontin in human aortic SMC along with vascular calcification and increased expression of PIT-1 [32,33].

BMP2, FGF2, PTH, TNF-α, IL-1β, and S100A12 (Table 2): In calcifying bovine and rat VSMC, an upregulation of the transcription factors CBFA1/RUNX2 and MSX2 and the phosphate cotransporter PIT-1 was described for BMP2 [42,43]. The pro-inflammatory RAGE ligand S100A12 induced CBFA1/RUNX2 and BMP2 expression in mouse VSMC [36], and an upregulation of the osteoblast markers CBFA1/RUNX2, osterix, and osteopontin was also shown in MSCs stimulated with the uremic toxins FGF2, PTH, TNF-α and IL-1β [41].

Indoxyl sulphate (Table 3): Parallel to inducing vascular calcification, indoxyl sulphate administration to human VSMC [30,51,52,53,54] or rats with hypertension [57] or 5/6 nephrectomy [51] increased the expression of CBFA1/RUNX2, ALP, BMP2 and osteopontin, while a reduction in α-SMA, SM22-α and/or SMTN expression could be observed. Recent in vitro data showed that indoxyl sulphate-induced osteogenic transdifferentiation and calcification of VSMC is mediated by the NF-κB and PI3K/AKT pathways [54].

#### 3.3.3. VSMC Apoptosis and Senescence

Increased apoptosis as well as cellular senescence of VSMC have been suggested as underlying mechanism of cardiovascular calcification. Cellular senescence goes along with increased vascular calcification and increased expression of osteogenic markers (RUNX2, BMP2) [65]. During the apoptosis process, apoptotic bodies are formed that are thought to contribute to vascular calcification through high calcium content and deposition in the extracellular matrix [65]. For the substances in Table 1, Table 2 and Table 3, the following links have been identified:

Phosphate (Table 1): In vitro, high phosphate treatment of human aortic VSMCs induced an increase in cellular apoptosis starting as soon as day 1 of culturing, as revealed by flow cytometry-mediated analysis of annexin V and propidium iodide staining [32].

Indoxyl sulphate (Table 3): As described above, Muteliefu et al. showed that, through increased ROS generation, indoxyl sulphate promotes a VSMC phenotype with increased expression of cell cycle arrest regulators and senescence markers (p53, p21, prelamin A) [55].

#### 3.3.4. Alkaline Phosphatase

Inorganic pyrophosphate (PPi) is a physicochemical inhibitor of hydroxyapatite crystal growth and a potential local as well as circulating inhibitor of cardiovascular calcification [68]. The enzyme ALP is able to hydrolyze PPi into phosphate ions [69]. An increase in ALP activity along with increased calcification in VSMC or MSC was shown for cytidine, threitol and urea [41] (Table 1); VCAM-1 [48], FGF2, PTH, TNF-α, and IL-1β [41] (Table 2), as well as for indoxyl sulphate [54] (Table 3).

## 4. Discussion

In CKD patients, uremic toxins accumulate due to failing kidney function, but current knowledge of the effect of uremic toxins on cardiovascular calcification is quite limited. Here, we systematically analyzed original research papers on uremic toxins in relation to cardiovascular calcification to examine whether and how uremic toxins may contribute to increased cardiovascular calcification in CKD.

In total, this review identified 29 uremic toxins (15 low molecular weight; 12 middle molecular weight; 2 protein-bound), upregulated in plasma of CKD patients, that have been studied for effects on cardiovascular calcification in in vitro, ex vivo or in vivo animal studies (Table 1, Table 2 and Table 3). For the 16 uremic toxins shown to increase vascular calcification, most could be directly linked to trigger increased VSMC osteogenic transdifferentiation or increased inflammation and oxidative stress (Figure 3). Additionally, the pro-inflammatory cytokines TNF-α and IL-1β as well as the pro-inflammatory S100A12 were shown to drive cellular transdifferentiation into an osteoblastic phenotype along with increasing calcification (Figure 3), directly linking inflammation to calcification over VSMC phenotype switching. Along the same line, indoxyl-sulphate as a protein-bound uremic toxin was shown to promote vascular calcification via inflammation (Figure 3). In addition to effects on VSMC transdifferentiation and inflammation, some substances were also identified to enhance ALP activity along with increasing vascular calcification (Figure 3).

Of the uremic toxins identified to impact on cardiovascular calcification (Table 1, Table 2 and Table 3), more were able to increase calcification (16x) than to decrease it (3x, being the guanidino compounds ADMA, GSA and SDMA) (Figure 2). Overall, this suggests that the uremic milieu in CKD patients shifts the balance to increased cardiovascular calcification. Among all uremic toxins studied, the harmful effect of protein-bound uremic toxin indoxyl sulphate in relation to cardiovascular calcification has been investigated most extensively (Table 3). Of note, for each middle molecular weight uremic toxin and protein-bound uremic toxin that was found to affect cardiovascular calcification, an increasing effect on calcification was revealed. This suggests that an efficient removal of both classes of uremic toxins may contribute to reduce cardiovascular calcification in CKD. However, while removing low molecular weight uremic toxins in CKD stage 5D patients using hemodialysis techniques is rather efficient, the removal of middle molecular weight toxins and especially protein-bound uremic toxins is still limited with current dialysis techniques [70]. For example, removal efficiency for the protein-bound toxins indoxyl sulphate and p-cresyl sulphate during a hemodialysis session was reported to reach only ~35% and 20–29%, respectively, independent of whether low-flux or high-flux membranes were used [71,72]. This is related to the formation of high molecular-weight complexes of these uremic toxins with plasma proteins, which prevents uremic toxin removal through the pores of the dialysis membranes [72]. Furthermore, the hydrophobic nature of these uremic toxins reduces their removal into hydrophilic dialysate. Applying alternative elimination principles in combination with classical filtration and diffusion technologies might be helpful to achieve a more effective removal of protein-bound uremic toxins in the future. This could include the adsorption of protein-bound uremic toxins [73,74,75,76,77], also in combination with fractionated plasma separation [77]. Furthermore, a high-frequency field could enhance the release of hydrophobic uremic toxins from plasma protein complexes and thereby increase their removal from blood during dialysis (patent numbers: WO2013004604A1, EP2729198A1, US20140246367A1). Along the same line, drugs such as ibuprofen have been tested for reducing the binding of protein-bound uremic toxins to plasma proteins, aiming to increase the unbound fraction of these toxins and thereby their removal efficiency during dialysis [78]. Alternative strategies aim at reducing the production of protein-bound uremic toxins in the gut through the use of probiotics, prebiotics, and synbiotics [79] or at reducing their absorption, e.g., by oral administration of the adsorbent AST-120 [80].

Despite the fact that, to date, over 140 uremic toxins have been described to be increased in CKD [20,21], only 29 uremic toxins were studied in relation to calcification in a mechanistic approach. In total, 12 other substances were only investigated for a potential association with the extent of cardiovascular calcification in CKD patients (Table S1). This, for example, also holds true for sclerostin, a regulator of bone metabolism with increased levels in serum and vasculature in CKD [81]. Whereas some studies could not detect a correlation between serum levels of sclerostin and the extent of cardiovascular calcification in patients in CKD stage 5D [82,83], others found a positive or negative correlation with calcification extent in CKD [81]. Sclerostin is a well-known inhibitor of bone formation by blocking the Wnt/beta-catenin pathway and suppressing osteoblast differentiation, but it currently remains unclear whether this sclerostin-Wnt axis also actively reduces vascular calcification [84]. This is not only important in the context of CKD, but also for the aging population, who similarly display increased medial calcification as well as increased sclerostin levels [81]. In addition, insights into a potential inhibitory role of sclerostin in cardiovascular calcification are essential in light of the current development of anti-sclerostin treatment for osteoporosis patients, which conceivably could contribute to increased vascular calcification and cardiovascular risk [81,84]. Thus, this systematic review suggests that investigating the impact of all uremic toxins on cardiovascular calcification systematically and in a standardized way could reveal those substances that would be most interesting for further research and, thereafter, potentially for drug development aiming to prevent or slow down the development and progression of cardiovascular calcification in CKD. Additionally, studying combinatorial and/or synergistic effects of uremic toxins are of importance as well, since previous studies revealed that combining blood levels of low molecular weight uremic toxins may help to improve the estimated survival of CKD stage 5D patients [85]. Preferentially, this should be done by a systematic approach, using standardized models for analyzing cardiovascular calcification in vitro and ex vivo [86].

Finally, in addition to direct effects of uremic toxins on processes impacting on cardiovascular calcification, indirect effects by uremic toxin-mediated post-translational modifications (PTMs) may also affect cardiovascular calcification. PTMs can critically affect protein functions and have been increasingly identified in CKD as well as CVD [87]. As one example, the uremic toxin cyanate as dissociation product of urea triggers carbamylation in CKD [88], with, for example, carbamylation of low-density lipoprotein shown to negatively influence endothelial function in CKD patients [89]. Thus, also uremic toxin-induced PTMs of calcification regulators may act as a link between CKD and increased risk of cardiovascular calcification. This has recently indeed been shown for protein carbamylation, which was revealed to promote calcification of VSMC by impairing mitochondrial function and inducing oxidative stress, thereby suppressing expression of ENNP1 as enzyme involved in PPi production [90]. This supports further research on PTMs in relation to vascular calcification.

Combined, an increased interdisciplinary focus on the mediators driving cardiovascular calcification in CKD as well as methodologies to interfere with calcification inducers is expected to contribute to the battle against increased cardiovascular calcification and mortality in CKD. Nonetheless, many challenges remain to be overcome: in addition to the identification of the main targets (uremic toxins and PTMs), we should focus on therapeutic translation. Here, different uremic toxin removal techniques, as discussed above, may have to be combined to enable a clinically relevant reduction in most protein-bound uremic toxins in CKD patients over extended time periods. Furthermore, although serum levels of, for example, p-cresyl sulphate and indoxyl sulphate have already been shown to positively correlate with vascular calcification and stiffness in CKD patients, [24,25,26] as well as with cardiovascular outcome in CKD [22,23,24,25], clinical studies will need to demonstrate whether reducing levels of such protein-bound uremic toxins indeed affects cardiovascular calcification and mortality in CKD patients.

In summary, this systematic review suggests that the uremic milieu in CKD patients shifts the balance to increased cardiovascular calcification. Of all middle molecular weight and protein-bound uremic toxins that were found to affect cardiovascular calcification, an increasing effect on calcification was revealed, demonstrating the need to focus on increased removal efficiency of these uremic toxin classes in current dialysis methods. Furthermore, this review clearly highlights that only a relatively small part of uremic toxins has been screened for effects on vascular calcification, supporting further efforts into a closer investigation of uremic toxins.

## Figures and Tables

**Table 1:** Low molecular weight substances increased in CKD and examined for causal effects on cardiovascular calcification or associated readouts.

Effect	Substance	Species	Tissue or Cell Type	Type of Study		Analysis of Calcification or Calcification-Associated Process * by	Reference	Plasma Level in CKD	Classified as Uremic Toxin **
				In Vitro	Animal				
INHIBITION	Asymmetric dimethylarginine (ADMA)	Human	VSMC	V ^$^		Alizarin red staining	Schepers [29]	↑ [§]	V
	Symmetric dimethylarginine (SDMA)	Human	VSMC	V ^$^		Alizarin red staining	Schepers [29]	↑ [§]	V
	Guanidinosuccinic acid (GSA)	Human	VSMC	V ^$^		Alizarin red staining	Schepers [29]	↑ [§]	V
INDUCTION	Phosphate (P)	Human	VSMC	V		Calcium content Alizarin red staining	Bouabdallah [30]	↑ [31]	
		Human	VSMC	V		Alizarin red staining Upregulation of MSX2, SOX9 and OPN Increased apoptosis	Cazana-Perez [32]		
		Human	VSMC	V		Calcium content Upregulation of CBFA1/RUNX2 and BMP2	Guerrero [33]		
		Human	VSMC	V		Alizarin red staining	Schepers [29]		
		Human	VSMC	V ^$^		High binding affinity of calcium to ECM	Shigematsu [34] ^1^		
		Rat	VSMC	V		Von Kossa staining	Shibata [35]		
		Rat	Aorta	V		Calcium content	Guerrero [33]		
		Mouse	Aorta		V ^2^	Von Kossa staining	Belmokhtar [36]		
		Mouse	VSMC	V		Alizarin red staining Increased ROS generation Upregulation of PIT-1, BMP2 and CBFA1/RUNX2	Belmokhtar [36]		
		Mouse	VSMC	V		Upregulation of BMP2 and OPN	Sage [37] ^1^		
		Rat	Aorta		V ^3^	Von Kossa staining	Shibata [35]		
	Calcium	Rat	Aorta	V		Calcium content	Azpiazu [38] ^1^	↑ [39]	
		Human	VSMC	V		Calcium content	Yang [40] ^1^		
	Cytidine	Human	MSC	V ^$^		ALP activity Calcium content	Hegner [41]	↑ [§]	V
	Urea	Human	MSC	V ^$^		ALP activity Calcium content	Hegner [41]	↑ [§]	V
	Threitol	Human	MSC	V ^$^		ALP activity Calcium content	Hegner [41]	↑ [§]	V
NO EFFECT	**Guanidino compounds:**	Human	VSMC	V		Alizarin red staining	Schepers [29]		
	ADMA							↑ [§]	V
	SDMA							↑ [§]	V
	GSA							↑ [§]	V
	Creatine							↑ [§]	V
	Creatinine							↑ [§]	V
	Guanidine							↑ [§]	V
	Guanidino acetic acid							↑ [§]	V
	Guanidino butyric acid							↑ [§]	V
	Guanidino propionic acid							↑ [§]	V
	Methylguanidine							↑ [§]	V

ALP—alkaline phosphatase; BMP2—bone morphogenetic protein 2; CBFA1/RUNX2—core-binding factor subunit alpha-1/runt-related transcription factor 2; CT—computed tomography; ECM—extracellular matrix; MSC–mesenchymal stromal cells (as progenitors of VSMC); MSX2—msh homeobox 2; OPN—osteopontin; PIT-1—sodium Pi co-transporter-1; ROS—reactive oxygen species; SOX9—SRY-box transcription factor 9; VSMC—vascular smooth muscle cells. * Readouts of calcification-associated processes, such as cell signaling and gene/protein expression, are shown in italic. [§] As described in comprehensive uremic toxins reviews [19,20]. ** As described in comprehensive uremic toxins reviews [19,20,21]. ^$^ In pro-calcifying medium. ^1^ Literature added manually after checking reference lists of included studies or recent reviews. ^2^ In vivo mouse model: 5/6 nephrectomy in Apoe^−/−^mice on a high- phosphorus diet vs. 5/6 nephrectomy in Apoe^-/-^ mice on a control diet for twelve weeks. ^3^ In vivo rat model: 5/6 nephrectomy in rats on a high- phosphorus and low-calcium diet vs. 5/6 nephrectomy in rats on a control diet for eight weeks; ↑ indicates increased plasma levels in CKD patients.

**Table 2:** Middle molecular weight substances increased in CKD and examined for causal effects on cardiovascular calcification or associated readouts.

Effect	Substance	Species	Tissue or Cell Type	Type of Study		Analysis of Calification or Calcification-Associated Process * by	Reference	Plasma level in CKD	Classified as Uremic Toxin **
				In vitro	Animal				
INDUCTION	Bone morphogenetic protein (BMP2)	Bovine	VSMC	V ^$^		Calcium content Upregulation of CBFA1/RUNX2	Chen [42]	↑ [42]	
		Rat	VSMC	V ^$^		Upregulation of CBFA1/RUNX2, MSX2 and PIT-1	Rong [43] ^1^		
	Fibroblast growth factor 2 (FGF2)	Human	MSC	V ^$^		ALP activity Alizarin red staining Calcium content Upregulation of CBFA1/RUNX2, OPN and osterix	Hegner [41]	↑ [§]	
	Interleukin-1β (IL-1β)	Human	MSC	V ^$^		ALP activity Alizarin red staining Calcium content Upregulation of CBFA1/RUNX2, OPN and osterix	Hegner [41]	↑ [§]	V
	Interleukin-8 (IL-8)	Human	VSMC	V ^$^		Alizarin red staining Calcium content Downregulation of OPN	Bouabdallah [30]	↑ [44]	
	Parathyroid hormone (PTH)	Human	MSC	V ^$^		ALP activity Alizarin red staining Calcium content Upregulation of CBFA1/RUNX2, OPN and osterix	Hegner [41]	↑ [§]	V
	S100A12 (RAGE ligand)	Mouse	VSMC	V		Alizarin red staining Increased ROS generation Upregulation of PIT-1, BMP2 and CBFA1/RUNX2	Belmokhtar [36]	↑ [45]	
	Salusin-β	Human	VSMC	V ^&^		Calcium content ALP activity Alizarin red staining Increased ROS generation Upregulation of BMP2 and CBFA1/RUNX2	Sun [46]	↑ [47]	
		Rat	Aorta	V ^&^		Calcium content ALP activity Von Kossa staining	Sun [46]		
	Tumor necrosis factor alpha (TNF-α)	Human	MSC	V		ALP activity Alizarin red staining Calcium content Upregulation of CBFA1/RUNX2, OPN and osterix	Hegner [41]	↑ [§]	V
		Human	VSMC	V ^$^		Calcium content	Guerrero [33]		
		Human	VSMC	V ^$^		ALP activity	Zickler [48]		
		Human	VSMC	V ^$^		Calcium content	Zickler [49]		
		Rat	Aorta	V ^$^		Calcium content	Guerrero [33]		
	Vascular cell adhesion molecule-1 (VCAM-1)	Human	VSMC	V ^$^		ALP activity Alizarin red staining	Zickler [48]	↑ [50]	
NO EFFECT	Soluble tumor necrosis factor receptor-1 (sTNFR-1)	Human	VSMC	V ^$^		ALP activity Alizarin red staining	Zickler [48]	↑ [48]	
	Soluble tumor necrosis factor receptor-2 (sTNFR-2)	Human	VSMC	V ^$^		ALP activity Alizarin red staining	Zickler [48]	↑ [48]	
	Soluble interleukin-2 receptor (sIL-2R)	Human	VSMC	V ^$^		ALP activity Alizarin red staining	Zickler [48]	↑ [48]	

ALP—alkaline phosphatase; BMP2—bone morphogenetic protein 2; CBFA1/RUNX2—core-binding factor subunit alpha-1/runt-related transcription factor 2; MSC—mesenchymal stromal cells (as progenitors of VSMC); OPN—osteopontin; PIT-1—sodium Pi co-transporter-1; ROS—reactive oxygen species; VSMC—vascular smooth muscle cells. * Readouts of calcification-associated processes, such as cell signaling and gene/protein expression, are shown in italic. [§] As described in comprehensive uremic toxins reviews [19,20]. ** As described in comprehensive uremic toxins reviews [19,20,21]. ^$^ In pro-calcifying medium. & In pro as well as non-calcifying media; ↑ indicates increased plasma levels in CKD patients. ^1^ Literature added manually after checking reference lists of included studies or recent reviews.

**Table 3:** Protein-bound substances increased in CKD and examined for causal effects on cardiovascular calcification or associated readouts.

Effect	Substance	Species	Tissue or Cell Type	Type of Study		Analysis of Calcification or Calcification-Associated Process * by	Reference	Plasma Level in CKD	Classified as Uremic Toxin **
				In vitro	Animal				
INDUCTION	Indoxyl sulphate (IS)	Human	VSMC	V ^$^		Calcium content Alizarin red staining Upregulation of CBFA1/RUNX2, ALP, BMP2 and OPN Downregulation of α-SMA and SM22-α	Bouabdallah [30]	↑ [§]	V
		Human	VSMC	V		Alizarin red staining Upregulation of CBFA1/RUNX2, ALP and OPN Downregulation of α-SMA and SMTN	Chen [51]		
		Human	VSMC		V	Alizarin red staining Upregulation of CBFA1/RUNX2 and OPN	Zhang [52]		
		Human	VSMC	V		Alizarin red staining Upregulation of CBFA1/RUNX2 and OPN	Chen [53]		
		Human	VSMC	V		Alizarin red staining ALP activity Downregulation of α-SMA Upregulation of CBFA1/RUNX2	He [54]		
		Human	VSMC	V		Upregulation of p53, p21, and prelamin A	Muteliefu [55]		
		Human	HepG2	V		Downregulation of fetuin-A	Ochi [56]		
		Rat	Aorta	V ^$^		Calcium content Alizarin red staining	Bouabdallah [30]		
		Rat	Aorta		V ^1^	Von Kossa staining Upregulation of OPN, CBFA1/RUNX2, ALP and osteocalcin	Adijiang [57]		
		Rat	Aorta		V ^2^	Von Kossa staining	Adijiang [58]		
		Rat	Aorta		V ^3^	Alizarin red staining Downregulation of α-SMA and SMTN Upregulation of CBFA1/RUNX2, ALP and OPN	Chen [51]		
		Rat	Aorta		V ^4^	Calcium content Von Kossa staining Activation of inflammation and coagulation pathways	Opdebeeck [59]		
		Rat	Aorta		V ^2^	Upregulation of 8-OHdG and MDA in the calcification area	Muteliefu [55]		
	p-Cresyl sulphate	Rat	Aorta		V ^4^	Calcium content Von Kossa staining Activation of inflammation and coagulation pathways	Opdebeeck [59]	↑ [§]	V

ALP—alkaline phosphatase; BMP2—bone morphogenetic protein 2; CBFA1/RUNX2—core-binding factor subunit alpha-1/runt-related transcription factor 2; MDA—malondialdehyde; 8-OHdG—8-hydroxyl-2’-deoxyguanosine; OPN—osteopontin; α-SMA—alpha-smooth muscle actin; SM22-α—smooth muscle protein 22-alpha; SMTN—smoothelin; VSMC—vascular smooth muscle cells. * Readouts of calcification-associated processes, such as cell signaling and gene/protein expression, are shown in italic. [§] As described in comprehensive uremic toxins reviews [19,20]. ** As described in comprehensive uremic toxins reviews [19,20,21]. ^$^ In pro-calcifying medium. ^1^ In vivo hypertensive rat model: (1) Dahl salt-resistant normotensive rats; (2) Dahl salt-sensitive hypertensive rats; (3) Dahl salt-sensitive hypertensive IS-administered rats for 30 weeks. ^2^ In vivo hypertensive rat model: (1) Dahl salt-resistant normotensive rats; (2) Dahl salt-resistant normotensive IS-administered rats; (3) Dahl salt-sensitive hypertensive rats; (4) Dahl salt-sensitive hypertensive IS-administered rats for 32 weeks. ^3^ In vivo uremic rat model: rats subjected to 5/6 nephrectomy were injected with IS at a dosage of 100 mg/kg/48 h for 24 weeks. Control rats with 5/6 nephrectomy received the same volume of phosphate-buffered saline injection every 48 h for 24 weeks. ^4^ In vivo uremic rat model: Male Wistar rats were exposed to a 10-day adenine sulphate treatment via daily oral gavage (600 mg/kg per day) to induce CKD. CKD rats fed a phosphate-enriched diet (1.2% Pi and 1.06% Ca) were randomly assigned to three treatment groups: (1) vehicle, (2) 150 mg/kg IS, or (3) 150 mg/kg p-cresyl sulphate; ↑ indicates increased plasma levels in CKD patients.

## Supplementary Materials

The following are available online at https://www.mdpi.com/2073-4409/9/11/2428/s1, Table S1: Substances studied for correlation with cardiovascular calcification extent in CKD patients.
