# Supplementary material for: Uremic Toxins Affecting Cardiovascular Calcification: A Systematic Review
Source: Cells. 2020 Nov 6;9(11):2428. doi: 10.3390/cells9112428 (PMC7694747; doi:10.3390/cells9112428)
Supplement: Supplementary file 1 [file cells-09-02428-s001.pdf]

Table S1: Substances studied for correlation with cardiovascular calcification extent in CKD patients.

Correlation with calcification	Type of substance	Substance	CKD patient cohort	Organ or tissue	Analysis of calcification by	Reference	Plasma level in CKD	Classified as uremic toxin**
POSITIVE	Low molecular weight substances	Phosphate (P)	Stage 3	Aorta, aortic- and mitral valve	Electron-beam CT or multi-detector CT	Adeney [1][1]	↑ [2]	
		Calcium-phosphate product (Ca x P)	Hemodialysis	Aorta	Plain X-rays and aortic arch calcification score	Shigematsu [3][1]		
	Middle molecular weight substances	Beta-2 microglobulin (B2M)	Stage 2-5D	Aorta	X-ray and CT scan	Liabeuf [4]	↑ [§]	V
		Fibroblast growth factor 23 (FGF23)	Stage 2-5D	Aorta	CT scan	Desjardins [5]	↑ [§]	V
			Stage 2-5D	Aorta	CT scan	Nasrallah [6]		
		Leptin	Stage 2-5D	Aorta	CT scan	de Oliveira [7]	↑ [§]	V
		Myoglobin (Mb)	Stage 2-5D	Aorta	CT scan	Lenglet [8]	↑ [§]	V
		Free light chains (FLCs) kappa (κ) and lambda (λ)	Stage 2-5D	Aorta	X-ray and CT scan	Desjardins [9]	↑ [§]	V
	Protein-bound substances	Indoxyl sulphate (IS)	Stage 2-5D	Aorta	CT scan	Barreto [10]	↑ [§]	V
			Hemodialysis	Coronary artery	CT scan	Asami [11]		
		p-Cresyl sulphate	Stage 2-5D	Aorta	X-ray and CT scan	Liabeuf [12]	↑ [§]	V
NO	Low molecular weight substances	Phosphate (P)	Peritoneal dialysis	Hands, pelvis	Plain X-rays and the Adragao scoring system	de Oliveira [13]	↑ [2]	
			Before renal transplantation	Mitral annulus	Echocardiography	Himalman [14]		
		Calcium (Ca)	Peritoneal dialysis	Hands, pelvis	Plain X-rays and the Adragao scoring system	de Oliveira [13]	↑ [15]	

			Before kidney transplantation	Mitral annulus	Echocardiography	Himalman [14]		
		Calcium-phosphate product (Ca x P)	Before kidney transplantation	Mitral annulus	Echocardiography	Himalman [14]		
	Middle molecular weight substances	Interleukin-6 (IL-6)	Stage 2-5D	Aorta	CT scan	Barreto [16]		
		Parathyroid hormone (PTH)	Before/after renal transplantation	Mitral annulus	Echocardiography	Himalman [14]		
		Sclerostin	Before renal transplantation, on dialysis	Hands, pelvis	Plain X-rays and the Adragao scoring system	de Oliveira [13]		
				Aorta	CT scan	Desjardins [17]		
		Bone alkaline phosphatase (BAP)	Before renal transplantation, on dialysis	Hands, pelvis	Plain X-rays and the Adragao scoring system	de Oliveira [13]		
		Total alkaline phosphatase (tAP)	Before renal transplantation, on dialysis	Hands, pelvis	Plain X-rays and the Adragao scoring system	de Oliveira [13]		
		Endotoxin (ET)	Stage 3-4	Superficial femoral artery	CT scan	John [18]		
	Protein-bound substances	Advanced glycation end products (AGEs)	Hemodialysis	Hands, pelvis	Plain X-rays and the Adragao scoring system	Franca [19]	↑ [§]	V

***CT** - computed tomography; [§] As described in comprehensive uremic toxins reviews [20,21]; ** As described in comprehensive uremic toxins reviews [20-22];* ↑ indicates increased plasma levels in CKD patients.

[1] Literature added manually after checking reference lists of included studies or recent reviews

References

1. Adeney, K.L.; Siscovick, D.S.; Ix, J.H.; Seliger, S.L.; Shlipak, M.G.; Jenny, N.S.; Kestenbaum, B.R. Association of serum phosphate with vascular and valvular calcification in moderate CKD. *J Am Soc Nephrol* **2009**, *20*, 381-387, doi:10.1681/ASN.2008040349.
2. Tertti, R.; Harmoinen, A.; Leskinen, Y.; Metsarinne, K.P.; Saha, H. Comparison of calcium phosphate product values using measurement of plasma total calcium and serum ionized calcium. *Hemodial Int* **2007**, *11*, 411-416, doi:10.1111/j.1542-4758.2007.00210.x.
3. Shigematsu, T.; Kono, T.; Satoh, K.; Yokoyama, K.; Yoshida, T.; Hosoya, T.; Shirai, K. Phosphate overload accelerates vascular calcium deposition in end-stage renal disease patients. *Nephrol Dial Transplant* **2003**, *18 Suppl 3*, iii86-89, doi:10.1093/ndt/gfg1022.
4. Liabeuf, S.; Lenglet, A.; Desjardins, L.; Neirynck, N.; Glorieux, G.; Lemke, H.D.; Vanholder, R.; Diouf, M.; Choukroun, G.; Massy, Z.A., et al. Plasma beta-2 microglobulin is associated with cardiovascular disease in uremic patients. *Kidney Int* **2012**, *82*, 1297-1303, doi:10.1038/ki.2012.301.
5. Desjardins, L.; Liabeuf, S.; Renard, C.; Lenglet, A.; Lemke, H.D.; Choukroun, G.; Drueke, T.B.; Massy, Z.A.; European Uremic Toxin Work, G. FGF23 is independently associated with vascular calcification but not bone mineral density in patients at various CKD stages. *Osteoporos Int* **2012**, *23*, 2017-2025, doi:10.1007/s00198-011-1838-0.

6. Nasrallah, M.M.; El-Shehaby, A.R.; Osman, N.A.; Fayad, T.; Nassef, A.; Salem, M.M.; Sharaf El Din, U.A. The Association between Fibroblast Growth Factor-23 and Vascular Calcification Is Mitigated by Inflammation Markers. *Nephron Extra* **2013**, *3*, 106-112, doi:10.1159/000356118.
7. de Oliveira, R.B.; Liabeuf, S.; Okazaki, H.; Lenglet, A.; Desjardins, L.; Lemke, H.D.; Vanholder, R.; Choukroun, G.; Massy, Z.A.; European Uremic Toxin Work, G. The clinical impact of plasma leptin levels in a cohort of chronic kidney disease patients. *Clin Kidney J* **2013**, *6*, 63-70, doi:10.1093/ckj/sfs176.
8. Lenglet, A.; Liabeuf, S.; Desjardins, L.; Neirynck, N.; Glorieux, G.; Lemke, H.D.; Vanholder, R.; Brazier, M.; Choukroun, G.; Massy, Z.A., et al. Prognostic implications of plasma myoglobin levels in patients with chronic kidney disease. *Int J Artif Organs* **2012**, *35*, 959-968, doi:10.5301/ijao.5000124.
9. Desjardins, L.; Liabeuf, S.; Lenglet, A.; Lemke, H.D.; Vanholder, R.; Choukroun, G.; Massy, Z.A.; European Uremic Toxin Work, G. Association between free light chain levels, and disease progression and mortality in chronic kidney disease. *Toxins* **2013**, *5*, 2058-2073, doi:10.3390/toxins5112058.
10. Barreto, F.C.; Barreto, D.V.; Liabeuf, S.; Meert, N.; Glorieux, G.; Temmar, M.; Choukroun, G.; Vanholder, R.; Massy, Z.A.; European Uremic Toxin Work, G. Serum indoxyl sulfate is associated with vascular disease and mortality in chronic kidney disease patients. *Clin J Am Soc Nephrol* **2009**, *4*, 1551-1558, doi:10.2215/CJN.03980609.
11. Asami, M.; Tanabe, K.; Ito, S.; Yoshida, E.; Aoki, J.; Tanimoto, S.; Horiuchi, Y.; Yoshida, M. Impact of Indoxyl Sulfate on Coronary Plaques in Patients on Hemodialysis. *Int Heart J* **2018**, *59*, 489-496, doi:10.1536/ihj.17-351.
12. Liabeuf, S.; Barreto, D.V.; Barreto, F.C.; Meert, N.; Glorieux, G.; Schepers, E.; Temmar, M.; Choukroun, G.; Vanholder, R.; Massy, Z.A., et al. Free p-cresylsulphate is a predictor of mortality in patients at different stages of chronic kidney disease. *Nephrol Dial Transplant* **2010**, *25*, 1183-1191, doi:10.1093/ndt/gfp592.
13. de Oliveira, R.A.; Barreto, F.C.; Mendes, M.; dos Reis, L.M.; Castro, J.H.; Britto, Z.M.; Marques, I.D.; Carvalho, A.B.; Moyses, R.M.; Jorgetti, V. Peritoneal dialysis per se is a risk factor for sclerostin-associated adynamic bone disease. *Kidney Int* **2015**, *87*, 1039-1045, doi:10.1038/ki.2014.372.
14. Himelman, R.B.; Helms, C.A.; Schiller, N.B. Is parathormone a cardiac toxin in uremia? *Int J Card Imaging* **1988**, *3*, 209-215, doi:10.1007/bf01797719.
15. Moe, S.M. Calcium as a cardiovascular toxin in CKD-MBD. *Bone* **2017**, *100*, 94-99, doi:10.1016/j.bone.2016.08.022.
16. Barreto, D.V.; Barreto, F.C.; Liabeuf, S.; Temmar, M.; Lemke, H.D.; Tribouilloy, C.; Choukroun, G.; Vanholder, R.; Massy, Z.A.; European Uremic Toxin Work, G. Plasma interleukin-6 is independently associated with mortality in both hemodialysis and pre-dialysis patients with chronic kidney disease. *Kidney Int* **2010**, *77*, 550-556, doi:10.1038/ki.2009.503.
17. Desjardins, L.; Liabeuf, S.; Oliveira, R.B.; Louvet, L.; Kamel, S.; Lemke, H.D.; Vanholder, R.; Choukroun, G.; Massy, Z.A.; European Uremic Toxin Work, G. Uremic toxicity and sclerostin in chronic kidney disease patients. *Nephrol Ther* **2014**, *10*, 463-470, doi:10.1016/j.nephro.2014.04.002.
18. John, S.G.; Owen, P.J.; Harrison, L.E.; Szeto, C.C.; Lai, K.B.; Li, P.K.; McIntyre, C.W. The impact of antihypertensive drug therapy on endotoxemia in elderly patients with chronic kidney disease. *Clin J Am Soc Nephrol* **2011**, *6*, 2389-2394, doi:10.2215/CJN.11211210.
19. Franca, R.A.; Esteves, A.B.A.; Borges, C.M.; Quadros, K.; Falcao, L.C.N.; Caramori, J.C.T.; Oliveira, R.B. Advanced glycation end-products (AGEs) accumulation in skin: relations with chronic kidney disease-mineral and bone disorder. *J Bras Nefrol* **2017**, *39*, 253-260, doi:10.5935/0101-2800.20170042.
20. Duranton, F.; Cohen, G.; De Smet, R.; Rodriguez, M.; Jankowski, J.; Vanholder, R.; Argiles, A.; European Uremic Toxin Work, G. Normal and pathologic concentrations of uremic toxins. *J Am Soc Nephrol* **2012**, *23*, 1258-1270, doi:10.1681/ASN.2011121175.
21. Vanholder, R.; De Smet, R.; Glorieux, G.; Argiles, A.; Baurmeister, U.; Brunet, P.; Clark, W.; Cohen, G.; De Deyn, P.P.; Deppisch, R., et al. Review on uremic toxins:

classification, concentration, and interindividual variability. *Kidney Int* **2003**, *63*, 1934-1943, doi:10.1046/j.1523-1755.2003.00924.x.

22. Vanholder, R.; Pletinck, A.; Schepers, E.; Glorieux, G. Biochemical and Clinical Impact of Organic Uremic Retention Solutes: A Comprehensive Update. *Toxins* **2018**, *10*, doi:10.3390/toxins10010033.
